# Alcoholism – can total abstinence be achieved or should we tread more lightly?

**DOI:** 10.1192/j.eurpsy.2023.1370

**Published:** 2023-07-19

**Authors:** D. M. Ciuperca

**Affiliations:** Psychiatry, “Dr Gh. Preda” Psychiatric Hospital, Sibiu, Romania

## Abstract

**Introduction:**

What defines any addiction, including alcohol dependence? Other than the accepted definitions from WHO, ICD or DSM, addiction is characterized by constant returns to alcohol consumption. Whether called relapse or lapse (slip), the truth is that patients - once abstinence is initiated - return to drinking in a matter of months (50% within 3 months, 65% within 6 months and 80% within 1 year). So why are we looking for total abstinence when treating patients with alcohol dependence, when the results are so poor? Should we look at this complex and intricated problem with monochrome lenses? Black or white? Couldn’t a more moderate approach present with better results? I think that a more reasonable response to this problem could be controlled drinking. Not just as an intermediate goal when seeking life long abstinence, but as a stand-alone indication, as an ultimate treatment goal. Are we looking to get healthy patients, with good social lives and satisfying quality of lives patients or are we just looking to obtain abstinence? Sure, alcohol should be forbidden to those with severe co-morbidities related to alcohol consumption (who could only worsen) should the consumption persist, but should all patients fall under the same category? Should total lifetime abstinence and relapse prevention remain the gold standard when treating this burden, even with emerging pharmacotherapy? Although additional pharmacotherapy for alcohol dependence exists, physicians may be reluctant to prescribe them, therefore they are severely underutilized. We should break barriers and rethink the way we treat this pathology, from medication to end-goals.

**Objectives:**

Raise awareness in the way we currently treat alcohol dependence among physicians, showing that a more considerate and moderate approach could be more beneficial, rather than a lifetime abstinence goal.

**Methods:**

Relevant papers were selected for review from literature, from both sides of the treatment approach spectrum

**Results:**

Although controlled drinking is accepted by a wide selection of physicians specializing in alcohol dependency treatment and studies have shown that pharmacotherapy for alcohol dependence works, current treatment guidelines (including EMA) still recommend total abstinence as an ultimate goal.

**Conclusions:**

The presentation is not intended to draw definitive conclusions, just raise awareness regarding the way we view and treat alcohol dependence.

**Disclosure of Interest:**

None Declared

